# Pubertal Testosterone Programs Adult Behavioral Adaptations to Sexual Experience through Infralimbic Cortex ΔFosB

**DOI:** 10.1523/ENEURO.0176-19.2019

**Published:** 2019-06-04

**Authors:** Kayla C. De Lorme, Nancy A. Staffend-Michael, Sarah C. Simmons, Alfred J. Robison, Cheryl L. Sisk

**Affiliations:** 1 Department of Psychology, Michigan State University, East Lansing, MI 48824; 2 Neuroscience Program, Michigan State University, East Lansing, MI 48824; 3 Department of Physiology, Michigan State University, East Lansing, MI 48824; 4 Department of Psychological Science, Gustavus Adolphus College, Saint Peter, MN 56082

**Keywords:** **Δ**FosB, ectopic mounts, medial prefrontal cortex, puberty, social proficiency, testosterone

## Abstract

Acquisition of social proficiency entails behavioral adaptations to social experience, including both behavioral flexibility and inhibition of behaviors inappropriate in specific social contexts. Here, we investigated the contributions of testosterone and ΔFosB, a transcription factor linked to experience-dependent neural plasticity, to the adolescent maturation of social proficiency in male-female social interactions. To determine whether pubertal testosterone organizes circuits underlying social proficiency, we first compared behavioral adaptations to sexual experience in male Syrian hamsters that were deprived of testosterone during puberty (prepubertal castration; NoT@P) to those of males deprived of testosterone for an equivalent period of time in adulthood (postpubertal castration; T@P). All males were given testosterone replacement in adulthood for two weeks before sexual behavior testing, where males were allowed to interact with a receptive female once per week for five consecutive weeks. T@P males showed the expected decrease in ectopic (mis-directed) mounts with sexual experience, whereas NoT@P males did not. In addition, sexual experience induced *FosB* gene products expression in the infralimbic cortex (IL) in T@P, but not NoT@P, males. Overexpression of ΔFosB via an adeno-associated viral (AAV) vector in the IL of NoT@P males prior to sexual behavior testing was sufficient to produce a behavioral phenotype similar to that of experienced T@P males. Finally, overexpression of ΔFosB in IL increased the density of immature spines on IL dendrites. Our findings provide evidence that social proficiency acquired through sexual experience is organized by pubertal testosterone through the regulation of ΔFosB in the IL, possibly through increasing synaptic lability.

## Significance Statement

Social proficiency is the ability to make experience-dependent behavioral adaptations that enhance the success of subsequent social interactions. In male rodents, social proficiency in adulthood is programmed by the pubertal rise in testosterone, but neuroendocrine mechanisms underlying this behavioral plasticity are not understood. We show that pubertal testosterone is necessary for both sexual proficiency and experience-dependent induction of ΔFosB in the infralimbic (IL) medial prefrontal cortex (mPfC) in adulthood. Furthermore, overexpression of ΔFosB in the IL increases immature dendritic spines on IL neurons and is sufficient to restore a socially proficient phenotype in males that lacked testosterone during puberty. Hormonal programming of experience-dependent regulation of prefrontal ΔFosB is a novel mechanism of adolescent development of behavioral and neural plasticity in adulthood.

## Introduction

A vital aspect of adolescent development is the acquisition of social behaviors and skills that prepare an individual for successful adult social interactions and promote evolutionary fitness. During adolescence, the primary social sphere transitions from family to peers, resulting in new social experiences and competencies (Nelson et al., 2005). Social proficiency is the ability of an individual to make experience-dependent behavioral adaptations that enhance the success of subsequent social interactions, and this proficiency involves behavioral flexibility and inhibition of maladaptive behaviors. Adolescent maturation of behavioral inhibition and social proficiency necessarily involves circuits underlying executive control of social motivation and learning (De Lorme et al., 2013), but the neural and endocrine mechanisms of this developmental change are largely unexplored; the present experiments were designed to identify these potential mechanisms.

Many of the behavioral changes related to the adolescent maturation of social proficiency have been attributed to puberty, which defines the onset of adolescence and is characterized by an increase in gonadal hormone secretion as reproductive maturation begins. The single-most important social interaction for evolutionary fitness is sexual behavior that leads to the production of offspring. Adolescent maturation of male sexual behavior is achieved in part through organizational effects of testosterone on the developing brain to program sexual proficiency, as shown in studies using male Syrian hamsters. For example, during a first sexual encounter with a receptive female, sexually naive adult male hamsters typically display a high rate of ectopic (mis-directed) mounts in addition to vaginally oriented mounts. With sexual experience, however, the number of ectopic mounts decreases, thereby improving sexual proficiency and reproductive success (Schulz and Sisk, 2006). Notably, the acquisition of sexual proficiency is not observed in adult hamsters deprived of testosterone during puberty via prepubertal castration; such males continue to show high rates of ectopic mounts even after repeated sexual encounters (Schulz and Sisk, 2006; De Lorme and Sisk, 2016). These data suggest that testosterone programs social proficiency by organizing neural circuitry involved in behavioral inhibition.

The medial prefrontal cortex (mPfC) and nucleus accumbens (NAc) are key components of the neural circuitry that regulates motivated behaviors. The mPfC is involved in behavioral flexibility and inhibition, whereas the NAc is critical for processing and evaluating social information and then generating a behavioral response (Sesack and Grace, 2010; Euston et al., 2012). Sexual experience induces long-term expression of the transcription factor ΔFosB within both the mPfC and NAc of male and female rodents, and experimental overexpression of ΔFosB in the NAc of sexually naive female and male rodents increases sexual performance and motivation (Wallace et al., 2008; Hedges et al., 2009; Pitchers et al., 2010). Therefore, ΔFosB induction within the mPfC and NAc appears to be an element of the restructuring of neural circuits that underlie long-term behavioral adaptations with sexual experience. One possible mechanism by which the induction of ΔFosB mediates experience-dependent plasticity is through the formation of immature dendritic spines, as ΔFosB overexpression in the NAc increases immature dendritic spines (Grueter et al., 2013; Robison et al., 2013; Eagle et al., 2015). Thus, ΔFosB may regulate behavioral plasticity by modulating transcription of downstream target genes related to synaptic plasticity.

Here, we investigated the neural mechanisms by which pubertal testosterone programs sexual proficiency in adulthood. First, to determine if pubertal testosterone affects regulation of ΔFosB, the effects of sexual experience on ΔFosB expression in the mPfC and NAc were compared in male hamsters that underwent adolescent development in either the presence or absence of testosterone during puberty. Next, to link ΔFosB with behavioral flexibility and inhibition, we determined whether overexpression of ΔFosB in the infralimbic cortex (IL) of the mPfC is sufficient to restore sexual proficiency in males that were deprived of testosterone during puberty. Finally, we asked whether sexual experience or overexpression of ΔFosB in the IL of sexually naive males induce similar changes in IL dendritic spines. We discovered that pubertal testosterone programs behavioral adaptability through the regulation of ΔFosB in the IL, and our data also suggest that ΔFosB in the IL may exert its behavioral effects through changes in glutamatergic synapse formation and/or stability.

## Materials and Methods

### General methods

#### Animals

**Figure 1.**
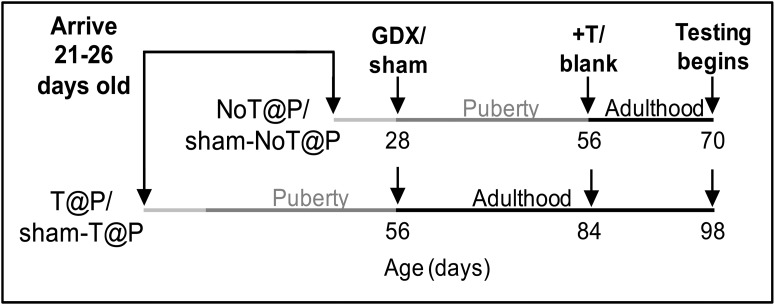
Experimental design of experiment 1. T@P and sham-T@P males arrived four weeks before NoT@P and sham-NoT@P males to control for the age of shipping and environment during puberty. Two to 7 d after the NoT@P and sham-NoT@P arrived, males were either GDX or sham-GDX during adulthood (P56; T@P and sham-T@P) or prepubertally (P28; NoT@P and sham-NoT@P). Four weeks later, T@P and NoT@P males received testosterone (T)-filled capsules and sham males received empty (blank) capsules of the same size. Sexual behavior testing began two weeks later.

To exclude age at shipment as a potentially confounding variable, and to ensure that postweaning housing conditions were similar for all experimental subjects in experiments 1 and 2, gonad-intact weanling [postnatal day (P)21–P26] male Syrian hamsters were ordered from Harlan Laboratories (Madison, WI). In addition, so that prepubertal and postpubertal gonadectomies, stereotaxic injections, behavioral testing, and tissue collection could be performed at the same time for all groups in experiments 1 and 2, the males that were gonadectomized (GDX) prepubertally were shipped and received four weeks later than males that were GDX postpubertally (Gonadectomy described below; Fig. 1). For experiment 3, sexually naive, gonad-intact adult male hamsters were ordered from Harlan. For all experiments, hamsters were individually housed in clear polycarbonate cages (30.5 × 10.2 × 20.3 cm) with ad libitum access to food and water in a 14/10 h light/dark cycle (lights out at 2 P.M.) on arrival. Sample sizes and experimental manipulations are described for each experiment below. Ovariectomized, sexually experienced female Syrian hamsters (four to seven months of age) from our colony (also originally obtained from Harlan) were used as stimulus animals in behavioral tests. All animals were treated in accordance with the National Institutes of Health Guide for the Care and Use of Laboratory Animals and protocols were approved by the Michigan State University Institutional Animal Care and Use Committee.

#### Gonadectomy

Gonadectomies were performed on male hamsters under isoflurane anesthesia and aseptic conditions. Before the procedure, ketoprofen analgesic (5 mg/kg, s.c.) was administered to each male. For the surgery, a bilateral scrotal incision 8–10 mm in length was made, the testes were then gently pulled through the incision, and the testicular veins and arteries were tied with suture silk (3–0) before removal of the testes. For prepubertal animals, testes were removed via cauterization of the testicular veins and arteries. For sham surgeries in experiment 1, a bilateral scrotal incision 8–10 mm in length was made, the testes were then gently pulled through the incision, and then placed back into the incision. After removal of the testes (gonadectomy) or replacement of testes back into the incision (sham surgery), the incisions were closed with suture silk (5–0).

#### Testosterone replacement

Two SILASTIC pellets (13 and 5 mm in length with 4 mm of sealing glue on both ends; inner diameter 1.98 mm; outer diameter 3.18 mm) that contained either testosterone or nothing were inserted subcutaneously through a 5-mm incision made on the dorsal midline between the scapulae of the animal while anesthetized with isoflurane. The pellets are constructed by loading SILASTIC tubing with powdered testosterone (or left empty) and sealing each end of the tube with surgical grade adhesive.

#### Stimulus females

Behavioral receptivity was induced in ovariectomized female hamsters by treatment with estradiol benzoate (10 µg in 0.05-ml sesame oil, s.c.) and progesterone (500 µg in 0.1-ml sesame oil, s.c.) 52 and 4–5 h, respectively, before use in sexual behavior tests with males. Each receptive female was used only once per sexual behavior test and was never paired with the same male more than once throughout the experiments.

#### Sexual behavior testing

In all experiments, testing began 1 h into the dark phase of the light-dark cycle under dim red light. Following a 2-min acclimation period in a clean large glass aquarium, each male was allowed to interact with a receptive female until the male achieved the sexual behavior criteria for that trial or after 30 min had passed, whichever came first. Previous experiments showed that during fixed-time tests, males deprived of testosterone during puberty achieve fewer intromissions and ejaculations than males deprived after puberty, and consequently, less sensory feedback that is potentially important for behavioral adaptations (Schulz et al., 2004; Schulz and Sisk, 2006). To address this possible confound, instead of absolute amount of time with the female, sexual behavior was equated for all males to ensure that any behavioral differences between groups were not due to different sensory experience. The behavioral criteria that ended each trial were: one ejaculation for trial 1, one ejaculation plus two intromissions for trial 2, two ejaculations for trial 3, one ejaculation plus two intromissions for trial 4, and one ejaculation for trial 5. The behavioral criteria were varied because we found in a previous study that when the same criterion was applied for each sexual behavioral test (achieving five intromissions) both gonad-intact and T@P male hamsters would decrease their sexual behavior after trial 3 (De Lorme and Sisk, 2016). Behavior testing was conducted once a week for five consecutive weeks (one of the five above-described trials per week). Males were excluded from further study if they did not meet the behavioral criteria in at least three out of the first four trials. Behavior during each trial was digitally recorded for later quantification.

#### Sexual behavior quantification

The behaviors investigated for experiments 1 and 2 were: rate (instances/min) of ectopic mounts (male grips female tightly and displays fast thrusting, but the mount is not vaginally oriented), latency to mount (male orients himself on the female’s hind flanks, grips her tightly with his forepaws, and displays fast thrusts), latency to intromit (male is vaginally-oriented and makes a long-lasting thrust resulting in vaginal penetration), latency to ejaculate (occurring after a series of intromissions followed by the male self-grooming and showing no sexual interest in the female for at least 20 s), and number of intromissions to reach ejaculation. Ejaculation latency and intromissions to ejaculate reflect sexual performance, whereas latencies to mount or intromit are measures of sexual motivation (Hull et al., 2002). Latencies to mount and intromit were timed from the moment the female was introduced to the male in the aquarium, and latency to ejaculate was defined as the amount of time that passed between the first intromission and ejaculation. Rate of ectopic mounting was used because males reached behavioral criteria to end the trial within varying times; rate was calculated by dividing the frequency of ectopic mounts displayed by the total test time per male per trial. All behavioral statistical analyses were performed using IBM SPSS software (version 19).

### Experiment 1: determine the effects of pubertal testosterone on sexual proficiency and ΔFosB expression in the mPfC and NAc

#### Animals (Fig. 1)

This experiment was conducted in three consecutive cohorts, with all experimental and control groups represented in each cohort. A total of 109 male hamsters were used in the experiment. Four to five weeks after the first group of weanling males arrived and 2–7 d after the second group of weanling males arrived (see above in the General methods Animals section for age at shipping), half of the first group (now adults, P56) and half of the second group (prepubertal, P28) males were either GDX (T@P and NoT@P, respectively) or received sham surgeries (sham-T@P and sham-NoT@P, respectively). The sham groups were used as age-matched methodological controls to confirm that behavioral differences found between T@P and NoT@P males were due to the presence or absence of testosterone during puberty, and not to age at the time of surgery or at the time of behavior testing. Four weeks after surgery, when all males were in adulthood, the T@P and NoT@P males received two testosterone-filled capsules and the sham-T@P and sham-NoT@P males received blank capsules of matched size. Two weeks later, approximately half of the males from each group began sexual behavior testing, while the other half of each group remained sexually naive. The sexually naive males were placed in an empty aquarium for 5 min before sexual behavior testing began for the sexually experienced males. This was done to ensure that the sexually naive males were not exposed to female pheromones that could have been present in the behavior testing room following sexual behavior testing, and eliminated any confound of handling and being placed in an aquarium, each of which could potentially influence the expression of ΔFosB in brain regions of interest. Thus, this design yielded eight experimental groups: (1) naive T@P, (2) experienced T@P, (3) naive NoT@P, (4) experienced NoT@P, (5) naive sham-T@P, (6) experienced sham-T@P, (7) naive sham-NoT@P, and (8) experienced sham-NoT@P.

#### Behavioral outliers and sample sizes

For each behavior, a box-plot using stem-and-leaf descriptives was used to identify the extreme data points within each experimental group. Dixon's Q-test was then used to determine if the extreme was a single statistical outlier. If the extreme was identified as an outlier for any behavior, the data for that animal were taken out of the analysis. One T@P male was an extreme high outlier for intromissions to ejaculation after sexual experience in experiment 1. Final sample sizes for each behavior analyzed are provided in the respective figures or figure legends.

#### Sexual behavior statistical analyses

Sexual behavior was analyzed using multilevel modeling (MLM), which provides an integrated assessment of experimental group (T@P, NoT@P) and/or sexual experience (trial 1: naive vs trial 5: experienced) on the measures of behavior described above in Sexual behavior quantification. The model treated the animal as the upper-level sampling unit and sexual experience as the lower-level sampling unit. Experimental groups (between-subjects variable) and sexual experience (trials 1 and 5, within-subject variable) were independent variables. The error structure was modeled to impose the traditional homoscedasticity assumption used in ANOVA. MLM provides a more powerful analysis than a traditional repeated measures ANOVA because it integrates non-independence between samples from the same subject in the model, and allows unequal sample sizes within the repeated measures. Analyses were performed separately for experimental and sham groups; *p* ≤ 0.05 was considered significant.

#### Tissue collection

Twenty-four hours after the final behavior test, 64 males (*n* = 8 per group, randomly chosen) were deeply anesthetized with an overdose of sodium pentobarbital (150 mg/kg, i.p.). The pan-FosB primary antibody used here for immunohistochemistry (rabbit α-FosB, sc-48 Santa Cruz Biotechnology) detects both FosB and ΔFosB; however, previous studies have confirmed that most full-length FosB is degraded within 18–24 h after stimulus (in this case either sexual behavior or being placed in an empty aquarium). Thus, the FosB-immunoreactive (-ir) cells are specifically enriched for ΔFosB when examined at the chosen time of 24 h relative to the behavioral manipulation (Perrotti et al., 2004, 2005, 2008; Wallace et al., 2008; Pitchers et al., 2010). Blood was collected via cardiac puncture, and the animals were perfused with 100 ml of buffered saline rinse and 150 ml of 4% paraformaldehyde. Brains were collected, postfixed overnight in 4% paraformaldehyde, and then stored in 20% sucrose until sectioning. Sections were cut (40 µm) into four coronal series using a cryostat and stored in cryoprotectant at –20°C until staining; one series was used for ΔFosB immunohistochemistry to identify, trace, and count cells in the cingulate (Cg1), prelimbic (PrL), and IL cortices of the mPfC and shell and core of NAc (ΔFosB immunohistochemistry described below).

#### Radioimmunoassay

In addition to cardiac blood collection from hamsters used for ΔFosB immunohistochemistry (described above in Tissue collection), trunk blood was collected from the rest of the hamsters (*n* = 5–7 per group), which were not perfused, via rapid decapitation 24 h after the final behavior test. Plasma from both blood collections was used to determine testosterone concentrations by radioimmunoassay. Plasma concentrations of testosterone were determined from duplicate 50-µl samples in a single assay using the Coat-A-Count Total Testosterone kit (Diagnostic Products). The intra-assay coefficient of variance was 3.5%, and the minimum limit of detectability was 0.12 ng/ml. All sexually naive and sexually experienced males within each experimental group had adult physiologic concentrations of circulating testosterone (Table 1).

**Table 1.** Concentrations of plasma testosterone

Plasma testosterone (ng/ml)		
Group	Sexual experience	
	Naive	Experienced
T@P	2.77 ± 1.01	3.54 ± 1.66
NoT@P	2.77 ± 1.21	3.68 ± 1.30
Sham-T@P	1.71 ± 0.72	2.59 ± 0.78
Sham-NoT@P	1.67 ± 0.88	2.57 ± 0.72

#### ΔFosB immunohistochemistry

Free floating sections were first rinsed 4 times for 5 min with 0.05 M Tris-buffered saline (TBS; pH 7.6) to remove cryoprotectant, and subsequently rinsed three times for 5 min with TBS between all incubations with reagents. Sections were exposed to 0.1% hydrogen peroxide for 10 min at room temperature to destroy endogenous peroxidases. The sections were then blocked in TBS containing 20% normal goat serum (NGS) and 0.3% Triton X-100 for 60 min. Sections were then incubated overnight at 4°C in 2% NGS and 0.3% Triton X-100 and the pan-FosB rabbit polyclonal antibody (1:10,000 dilution for a final concentration of 0.02 µg/ml; sc-48 Santa Cruz Biotechnology). After primary antibody incubation, the sections were washed in TBS, and then incubated for 1 h in goat anti-rabbit horseradish peroxidase-conjugated secondary antibody (1:500 dilution) containing 2% NGS and 0.3% Triton X-100 in TBS. Then, the sections were incubated in Vectastain ABC Elite kit (Vector) for 1 h at room temperature before visualizing the immunoreactivity with diaminobenzidine (DAB; 0.5 mg/ml plus NiCl with 0.025% H_2_O_2_). The sections were rinsed in TBS four times before mounting them onto glass slides. The mounted sections were then put through a series of ethanols and xylene before coverslipping.

#### Immunohistochemistry analysis

The number of ΔFosB-ir cells was quantified in 3 anatomically matched sections for both mPfC and NAc. For the mPfC, a 450 × 450-µm box was placed in each subregion (Cg1, Prl, IL) relative to brain midline and corpus callosum landmarks, and for the NAc, two 250 × 250-µm boxes were placed in the NAc core and a 250 × 250-µm box in the NAc shell relative to anterior commissure and lateral ventricle. The Morin and Wood (2001) hamster atlas was also used as a reference. Box placements were determined bilaterally under a 4× objective.

Cell counts were made within each contour by an experimenter blind to treatment group with an UPlanSApo 40× (0.9NA) objective. Cells were considered ΔFosB-ir if they had a distinct nucleus with visible puncta stained opaque, dark purple-blue; cells that had translucent, lighter stained nuclei were not counted. Sample images of stained cells were also used as a guide to determine which cells met the criteria described above. All analyses were performed on an Olympus BX51 microscope under brightfield illumination using Neurolucida (version 9; Microbrightfield). The number of ΔFosB-ir cells from the three tissue sections per subregion per hamster was used in statistical analysis (described below).

#### ΔFosB-ir expression statistical analysis

To provide an integrated assessment of pubertal testosterone and sexual experience on ΔFosB-ir expression within the mPfC and NAc, MLM was used. For the analysis, the model treated the animal as the upper-level sampling unit and tissue section as the lower-level sampling unit, with pubertal testosterone and sexual experience as independent variables and ΔFosB-ir cell number as the dependent variable. Interactions were followed up by MLMs within a subset of animals, as appropriate. All statistical analyses were performed using IBM SPSS software (version 19). Analysis was performed separately for experimental and sham groups; *p* ≤ 0.05 was considered significant. Due to poor tissue quality for some males, the sample sizes varied between groups of animals. Final sample sizes for each brain region are provided in the data figures.

### Experiment 2: determine whether overexpression of ΔFosB in the IL is sufficient to restore sexual proficiency in NoT@P males

Experiment 1 showed that the absence of testosterone during puberty impaired sexual proficiency: NoT@P males continued to show high rates of ectopic mounts even after sexual experience. In addition, sexual experience led to an increase in ΔFosB expression in the IL mPfC and NAc core only in T@P males. Experiment 2 was designed to probe the role of ΔFosB in sexual proficiency by asking whether overexpression of ΔFosB in the IL of NoT@P males is sufficient to instate sexual proficiency. We chose to overexpress ΔFosB in the IL instead of NAc core because the impairment in sexual proficiency seen in NoT@P males appears to be related to impairment in behavioral inhibition, which is more closely linked to IL function than to NAc core function (Vertes, 2006; Euston et al., 2012).

#### Animals

Thirty-four male hamsters were used for this experiment; they arrived as gonad-intact weanlings in two groups four weeks apart, as in experiment 1. Four to five weeks after the first group arrived and 2–7 d after the second group arrived, the first group (now adults P56; T@P) and the second group (prepubertal P28; NoT@P) males were GDX. Four weeks later, under isoflurane anesthesia, all of the T@P (P84) and NoT@P (P56) males received two testosterone-filled capsules and bilateral microinjections of recombinant adeno-associated viral (rAAV) vectors encoding either green fluorescence protein (GFP) or GFP and wild-type ΔFosB aimed at the IL. Thus, there were three groups: T@P-GFP, NoT@P-GFP, and NoT@P-ΔFosB. For the microinjections, a small hole was drilled in the skull and a 5-μl Hamilton syringe (26-gauge, Hamilton) was lowered at a 20° angle to the level of the IL (3.3 mm rostral, ±1.6 mm lateral, and 4.5 mm ventral relative to bregma) based on the Morin and Wood (2001) atlas. The syringe was kept in place for 2 min before injections and then either rAAV-ΔFosB or rAAV-GFP (1.0 µl per hemisphere) was injected into the IL over 10 min, with the syringe kept in place for an additional 5 min after injection was complete. AAV2/5 viral vectors were purchased from the University of North Carolina Viral Vector Core, and titres were ∼1.0 × 10^13^ transducing particles per microliter. These AAV vectors reach maximal expression around 10 d and sustain expression indefinitely (Vialou et al., 2010; Eagle et al., 2015; Sarno and Robison, 2018). These vectors only infect neurons and are no more toxic than vehicle alone (Zachariou et al., 2006). This AAV overexpression methodology provides the advantages of temporal (in this case, adulthood) and spatial (in this case, IL neurons) specificity, and the disadvantages of a greater level of expression than is typically seen in neurons and expression throughout the neuron, rather than specifically in the nucleus (Vialou et al., 2010; Robison et al., 2013; Eagle et al., 2015). As no other strategy for testing the role of ΔFosB in hamsters is currently available, we feel the advantages of viral overexpression outweigh the disadvantages, and this approach can yield critical new understanding of the molecular mechanisms of hamster behavior. Two weeks later (P70 and P98, respectively), all of the males from each group underwent sexual behavior testing over the next five weeks, as described in General Methods above.

#### Viral vector placement, exclusion criteria, and sample sizes

Based on the previously described behavioral outlier criteria for experiment 1, one NoT@P-ΔFosB male was an extreme high outlier for ectopic mounts per minute after sexual experience. Additionally, males were excluded from behavioral analysis if injections were misplaced or the viral vector was not expressed (*n* = 8), or if they displayed impaired motor behavior (e.g., difficulty walking or slowed movement) following stereotaxic surgery (*n* = 2). The GFP control males were included in analyses if the overexpression was located within any subregion of the mPfC, whereas NoT@P-ΔFosB males were included only if the overexpression was located bilaterally in the IL. Of the five NoT@P-ΔFosB males removed from analysis, three of them had evidence of viral vector expression in other regions of the brain (two in the Cg1/posterior PrL and one in the secondary motor cortex), while the other two males did not show any evidence of viral vector expression. Thus, a total of 10 males were excluded from behavioral analysis yielding a total of 24 males, with T@P-GFP *n* = 7, NoT@P-GFP *n* = 10, and NoT@P- ΔFosB *n* = 7. It should be noted that in four out of the seven NoT@P-ΔFosB group, overexpression of ΔFosB occurred in both the IL and the ventral region of the PrL. Final sample sizes for each behavior analyzed are provided in the respective figures or figure legends.

#### Sexual behavior statistical analysis

Statistical analysis for sexual behavior in experiment 2 was the same as previously described for experiment 1. In addition, the nature of the main effect of experimental group (T@P-GFP, NoT@P-GFP, NoT@P- ΔFosB) was determined using a Bonferroni correction, and interactions were followed up by one-way ANOVAs for between subject measures or MLM for repeated measures within a subset of animals, as appropriate. *p* ≤ 0.05 was considered significant.

#### Tissue processing

Twenty-four hours after the final behavior test, all hamsters were deeply anesthetized with an overdose of sodium pentobarbital (150 mg/kg, i.p.). Blood was collected via cardiac puncture, and the animals were perfused with 100 ml of buffered saline rinse and 150 ml of 4% paraformaldehyde. Brains were collected, postfixed overnight in 4% paraformaldehyde, and then stored in 20% sucrose until sectioning. To verify the correct placement of the injection using GFP as a marker, sections were cut into 40-µm coronal sections using a cryostat and stored in cryoprotectant for 30–60 min until mounting. The sections were then washed in TBS, mounted, and coverslipped while still wet with Vectashield hard set mounting medium (Vector Laboratories). The ΔFosB vector contains a segment expressing GFP, allowing for the injection site and extent of infection of cells to be verified by GFP visualization using an Olympus BX51 microscope under fluorescence illumination.

#### Radioimmunoassay

Plasma from blood collection was used to determine testosterone concentrations by radioimmunoassay as described above for Experiment 1. The intra-assay coefficient of variance was 5.4% and the minimum limit of detectability was 0.11 ng/ml. All males had adult physiologic concentrations of circulating testosterone, with no significant differences among the groups (T@P-GFP 4.07 ± 1.16 ng/ml, NoT@P-GFP 4.76 ± 0.85 ng/ml, NoT@P-ΔFosB 3.84 ± 0.73 ng/ml).

### Experiment 3: determine the effects of sexual experience and overexpression of ΔFosB in IL on dendritic spine number and morphology

Experiments 1 and 2 provided evidence that pubertal testosterone programs sexual proficiency through the regulation of ΔFosB in the IL. To explore a possible mechanism through which ΔFosB in the IL mediates this experience-induced plasticity, experiment 3 investigated whether sexual experience and overexpression of ΔFosB in IL have similar effects on IL dendritic spine number and morphology, as has been reported for sexual experience and ΔFosB in NAc (Pitchers et al., 2013).

#### Animals

A total of 10 gonad-intact adult male hamsters were used for this experiment. Stereotaxic surgery and sexual behavior testing were performed as described in experiment 2. Hamsters were randomly assigned to one of three groups to investigate the effects of sexual experience and ΔFosB overexpression on spine density in the IL: sexually naive plus GFP (naive-GFP; *n* = 4), sexually experienced (experienced-GFP; *n* = 3), and sexually naive plus ΔFosB (naive-ΔFosB; *n* = 3) males. AAV injections were performed as previously described in experiment 2. Twenty-one days later, males in the experienced-GFP group underwent five weeks of sexual behavior experience as described above in General methods Sexual behavior testing; males in the naive-GFP and naive-ΔFosB groups were placed in clean empty glass aquaria for 5 min in lieu of a sexual behavior experience.

#### Tissue collection and processing

Twenty-four hours after the final behavior test, all hamsters were deeply anesthetized with an overdose of sodium pentobarbital (150 mg/kg, i.p.). Animals were then transcardially perfused with 100-ml ice-cold PBS followed by 150-ml 4% paraformaldehyde. Brains were postfixed 24 h in 4% paraformaldehyde and cryopreserved in 20% sucrose. Brains were cut into 100 µm sections on a Vibratome 3000 EP (Leica Microsystems) and rinsed in PBS. Free-floating immunofluorescent staining was performed using a goat anti-GFP primary antibody (ab5450, 1:1000, Abcam) followed by an Alexa Fluor 488 secondary antibody (705-545-147, 1:200, Jackson ImmunoResearch). Sections were mounted on slides using DPX mounting medium (Sigma-Aldrich).

#### Dendritic spine quantification

GFP/Alexa Fluor 488 fluorescence was visualized using an Olympus FluoView 1000 Filter-based Laser Scanning Confocal Microscope with the z-step size of 0.5 μm and numerical aperture of 1.40 using a 100× lens. Spines were on the dendritic arbors of cortical pyramidal neurons in IL Layers III, V, and VI, and analyzed essentially as previously described (Christoffel et al., 2011). Briefly, dendritic segments 50–150 μm away from the soma were randomly chosen from IL neurons that expressed GFP. Z-stack images were acquired for reconstruction and morphologic analysis using NeuronStudio with the rayburst algorithm. NeuronStudio classifies spines as thin, mushroom, or stubby based on the following values: (1) aspect ratio, (2) head to neck ratio, and (3) head diameter. Spines with a neck can be classified as either thin or mushroom, and those without a significant neck are classified as stubby. Spines with a neck are labeled as thin or mushroom based on head diameter.

#### Dendritic spine statistical analysis

The three groups were analyzed for differences in dendritic spine density by one-way ANOVA followed by Bonferroni *post hoc* test for pairwise differences between individual groups. In this model, the unit of analysis was the number of dendrites within each animal group, yielding sample sizes as: naive-GFP, 22; experienced-GFP, 21; and naive-ΔFosB, 22; *p* ≤ 0.05 was considered significant. Statistical analysis was performed using IBM SPSS software (version 19).

## Results

Statistics for all results are reported in respective tables.

### Sham controls: age at surgery does not affect sexual behavior or ΔFosB in the mPfC

Sham controls were used to confirm that any differences we found between T@P and NoT@P males are due to hormonal manipulation and not age of testing or surgery. The sham-T@P and sham-NoT@P males were tested at the same time and using the same protocol as experiment 1. There were no significant differences between the two sham groups for any of the behaviors (data not shown). In both groups, latency to mount, latency to intromit, latency to ejaculate, and intromissions to ejaculation all significantly decreased with sexual experience (Table 2). Additionally, the two sham groups did not differ in overall expression of ΔFosB in the mPfC subregions analyzed, and sexual experience induced ΔFosB in the IL in both sham groups (Table 2). These findings from the sham groups indicate that differences in behavioral or neural measures observed between T@P and NoT@P males are due to the presence or absence of testosterone during puberty, and not to age at the time of surgery or at the time of behavior testing. Results from sham groups will not be further discussed nor represented in the figures.

**Table 2.** Statistics for sham controls

Figure	Independent variable(s)	Dependent variable	Statistics (*significant)
NA	Sexual experience	Latency to mount	*F*_(1,24)_ = 14.65; *p* = 0.001*
		Latency to intromit	*F*_(1,24)_ = 14.33; *p* = 0.001*
		Latency to ejaculate	*F*_(1,24)_ = 31.27; *p* = 0.001*
		Intromissions to ejaculation	*F*_(1,17)_ = 12.24; *p* = 0.003*
		ΔFosB in the IL	*F*_(1,23)_ = 4.45; *p* = 0.046*

**Table 3.** Statistics for experiment 1

Figure	Independent variable(s)	Dependent variable	Statistics (*significant)	*Post hoc* comparison (if appropriate)	*Post hoc* statistics (*significant)
2	Pubertal testosterone	Ectopic mounting	*F*_(1,27)_ = 6.75; *p* = 0.015*		
	Sexual experience		*F*_(1,27)_ = 0.69; *p* > 0.05		
	Pubertal testosterone × sexual experience		*F*_(1,27)_ = 0.54; *p* > 0.05		
3	Pubertal testosterone	Latency to mount	*F*_(1,27)_ = 0.272; *p* > 0.05		
	Sexual experience		*F*_(1,27)_ = 17.43; *p* < 0.001*		
	Pubertal testosterone × sexual experience		*F*_(1,27)_ = 0.309; *p* > 0.05		
	Pubertal testosterone	Latency to intromit	*F*_(1,27)_ = 1.01; *p* > 0.05		
	Sexual experience		*F*_(1,27)_ = 15.84; *p* < 0.001*		
	Pubertal testosterone × sexual experience		*F*_(1,27)_ = 2.50; *p* > 0.05		
	Pubertal testosterone × sexual experience	Latency to ejaculate	*F*_(1,27)_ = 12.70; *p* = 0.001*	Pubertal testosterone in naive males	*F*_(1,24)_ = 10.33; *p* = 0.004*
				Pubertal testosterone in experienced males	*F*_(1,27)_ = 0.641; *p* > 0.05
	Pubertal testosterone	Intromissions to ejaculation	*F*_(1,26)_ = 0.465; *p* > 0.05		
	Sexual experience		*F*_(1,26)_ = 25.00; *p* < 0.001*		
	Pubertal testosterone × sexual experience		*F*_(1,26)_ = 2.73; *p* > 0.05		
4*D*	Pubertal testosterone × sexual experience	ΔFosB in the IL	*F*_(1,23)_ = 10.86; *p* = 0.003*	sexual experience in T@P males	*F*_(1,12)_ = 14.06; *p* = 0.003*
			*** ***	sexual experience in NoT@P males	*F*_(1,11)_ = 0.721; *p* > 0.05
	Pubertal testosterone	ΔFosB in the Cg1	*F*_(1,23)_ = 0.046; *p* > 0.05		
	Sexual experience		*F*_(1,23)_ = 0.082; *p* > 0.05		
	Pubertal testosterone × sexual experience		*F*_(1,23)_ = 0.66; *p* > 0.05		
	Pubertal testosterone	ΔFosB in the PrL	*F*_(1,23)_ = 0.133; *p* > 0.05		
	Sexual experience		*F*_(1,23)_ = 2.04; *p* > 0.05		
	Pubertal testosterone × sexual experience		*F*_(1,23)_ = 2.57; *p* > 0.05		
5*C*	Pubertal testosterone × sexual experience	ΔFosB in the NAc core	*F*_(1,23)_ = 5.42; *p* = 0.029*	Sexual experience in T@P males	*F*_(1,12)_ = 9.66; *p* = 0.009*
				Sexual experience in NoT@P males	*F*_(1,11)_ = 0.042; *p* > 0.05
	Pubertal testosterone	ΔFosB in the NAc shell	*F*_(1,23)_ = 1.60; *p* > 0.05		
	Sexual experience		*F*_(1,23)_ = 7.041; *p* = 0.014*		
	Pubertal testosterone × sexual experience		*F*_(1,23)_ = 2.68; *p* > 0.05		

**Table 4.** Statistics for experiment 2

Figure	Independent variable(s)	Dependent variable	Statistics (*significant)	*Post hoc* comparison (if appropriate)	*Post hoc* statistics (*significant)
7	ΔFosB overexpression	Ectopic mounting	*F*_(2,22)_ = 3.94; *p* = 0.035*	NoT@P-ΔFosB vs NoT@P-GFP males	*p* = 0.043*
	Sexual experience		*F*_(1,21)_ = 1.25; *p* > 0.05		
	ΔFosB overexpression × sexual experience		*F*_(1,21)_ = 1.20; *p* > 0.05		
8	ΔFosB overexpression × sexual experience	Latency to mount	*F*_(2,19)_ = 3.61; *p* = 0.047*	Sexual experience in NoT@P-ΔFosB males	*F*_(1,6)_ = 11.00; *p* = 0.015*
				Sexual experience in T@P-GFP males	*F*_(1,6)_ = 1.61; *p* > 0.05
				Sexual experience in NoT@P-GFP males	*F*_(1,7)_ = 0.61; *p* > 0.05
	ΔFosB overexpression	Latency to intromit	*F*_(2,20)_ = 1.12; *p* > 0.05		
	Sexual experience		*F*_(1,20)_ = 14.09; *p* = 0.001*		
	ΔFosB overexpression × sexual experience		*F*_(2,20)_ = 3.06; *p* > 0.05		
	ΔFosB overexpression	Latency to ejaculate	*F*_(2,20)_ = 2.23; *p* > 0.05		
	Sexual experience		*F*_(1,21)_ = 15.20; *p* < 0.001*		
	ΔFosB overexpression × sexual experience		*F*_(2,20)_ = 1.64; *p* > 0.05		
	ΔFosB overexpression	Intromissions to ejaculation	*F*_(2,21)_ = 0.87; *p* > 0.05		
	Sexual experience		*F*_(1,20)_ = 11.70; *p* = 0.003*		
	ΔFosB overexpression × sexual experience		*F*_(2,20)_ = 0.06; *p* > 0.05		

**Table 5.** Statistics for experiment 3

Figure	Independent variable(s)	Dependent variable	Statistics (*significant)	*Post hoc* comparison (if appropriate)	*Post hoc* statistics (*significant)
9*B*	Experimental group (naive-GFP, experienced-GFP, naive-ΔFosB )	Thin spines	*F*_(2,62)_ = 1.717; *p* < 0.001*	Naive-ΔFosB vs naive-GFP males	*p* < 0.001*
			* *	Naive-ΔFosB vs experienced-GFP males	*p* = 0.016*
	Experimental group (naive-GFP, experienced-GFP, naive-ΔFosB )	Stubby spines	*F*_(2,62)_ = 0.723; *p* > 0.05		** **
	Experimental group (naive-GFP, experienced-GFP, naive-ΔFosB )	Mushroom spines	*F*_(2,61)_ = 4.080; *p* = 0.022*	Naive-GFP vs experienced-GFP males	*p* = 0.029*
	Experimental group (naive-GFP, experienced-GFP, naive-ΔFosB )	Total spines	*F*_(2,62)_ = 9.359; *p* < 0.001*	Naive-ΔFosB vs naive-GFP males	*p* < 0.001*

### Experiment 1: pubertal testosterone programs behavioral adaptations to sexual experience and experience-dependent expression of ΔFosB in the mPfC and NAc (Table 3)

**Figure 2.**
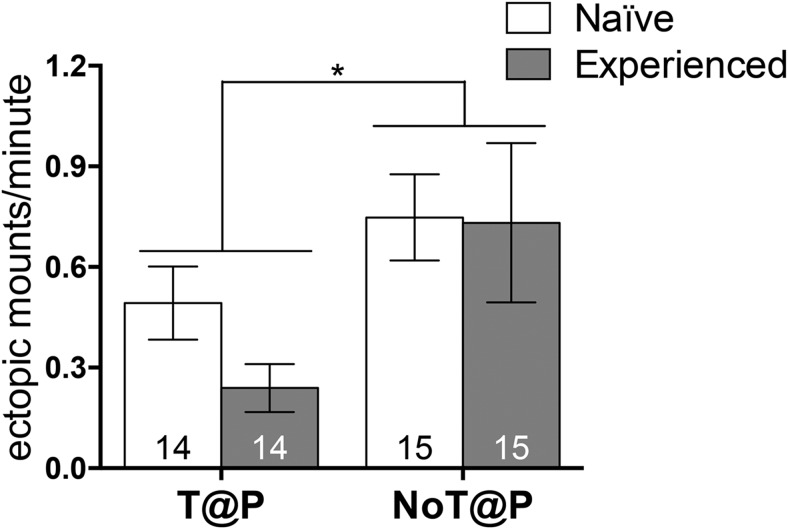
Rate of ectopic mounting is dependent on pubertal testosterone. T@P males had significantly fewer ectopic mounts per minute compared to NoT@P males. Bars represent mean (±SEM); numbers on bars indicate sample size. *Main effect of pubertal testosterone, *p* ≤ 0.05.

**Figure 3.**
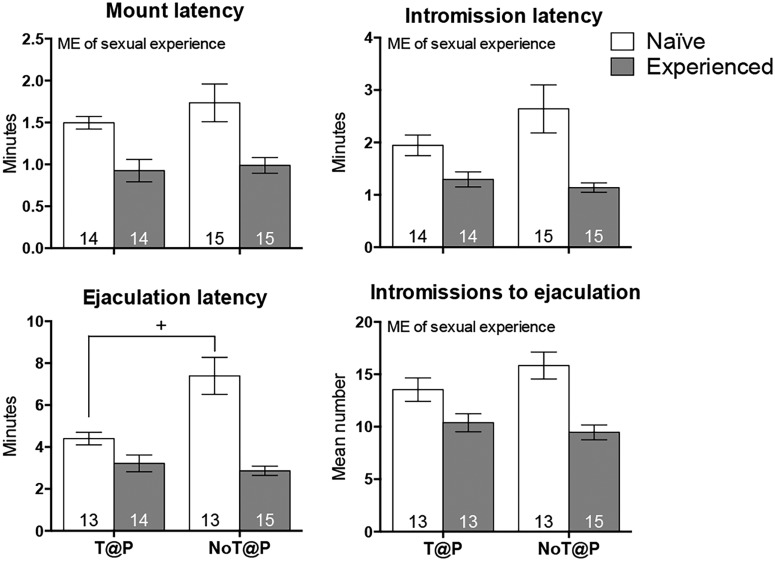
The effects of pubertal testosterone and sexual experience on latency to mount, intromit, and ejaculate and number of intromissions to ejaculation. Mount latency: there was a main effect (ME) of sexual experience on mount latency with sexually experienced males having shorter latencies to mount compared to sexually naive males. Intromission latency: There was an ME of sexual experience on intromission latency with sexually experienced males having shorter latencies to intromit compared to sexually naive males. Ejaculation latency: there was a pubertal testosterone × sexual experience interaction on ejaculation latency with sexually naive NoT@P males having a longer latency to ejaculate compared to sexually naive T@P males. This effect was not seen in sexually experienced males. Intromissions to ejaculate: there was an ME of sexual experience for intromissions to ejaculate with sexually experienced males having less intromissions to achieve ejaculation compared to sexually naive males. Bars represent mean (±SEM); numbers on bars indicate sample size. +Interaction between pubertal testosterone and sexual experience, *p* ≤ 0.05.

The absence of testosterone during puberty led to significantly higher overall rates of ectopic mounting in NoT@P males compared with T@P males, independent of sexual experience (Fig. 2). Although the pubertal testosterone × sexual experience interaction was not statistically significant, only T@P males showed a clear decrease in the rate of ectopic mounting with sexual experience (naive mean ± SEM rate: 0.492 ± 0.156, experienced mean ± SEM rate: 0.239 ± 0.156) compared to NoT@P males (naive mean ± SEM rate: 0.748 ± 0.151, experienced mean ± SEM rate: 0.732 ± 0.151). In contrast, the latency to mount, latency to intromit, and number of intromissions to reach ejaculation were all significantly reduced by sexual experience (Fig. 3), independent of pubertal testosterone. There was an interaction between sexual experience and pubertal testosterone for ejaculation latency because latency to ejaculate was much higher in naive NoT@P males compared with naive T@P males (Fig. 3).

**Figure 4.**
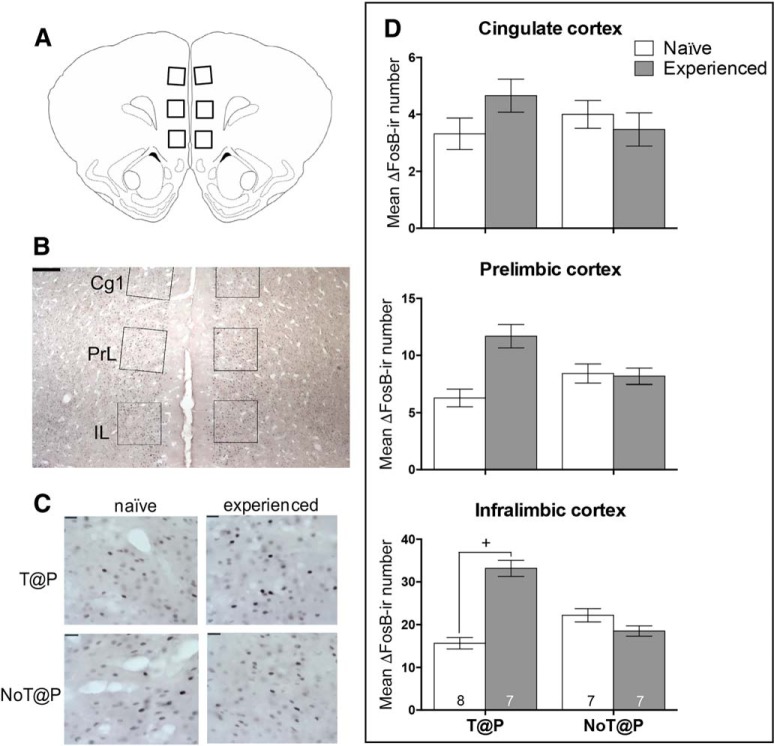
Number of ΔFosB-ir cells in IL is dependent on pubertal testosterone and sexual experience. ***A***, Brain atlas (Morin and Wood, 2001) representation of a coronal section containing the mPfC. ***B***, Photomicrographs of drawn contours of the mPfC onto immunohistochemically-treated tissue sections at 4× objective. The mPfC included the anterior Cg1, PrL, and IL cortices; scale bar = 250 µm. ***C***, The 2 × 2 panel of photomicrographs below the bar graph are representative images of ΔFosB-ir in the IL for the specified group of males; scale bars = 25 µm. ***D***, In the CgL and PrL, there were no effects or interactions of pubertal testosterone and sexual experience on ΔFosB-ir cells, respectively. In the IL, there was an interaction between pubertal testosterone and sexual experience on ΔFosB-ir cells with sexual experienced T@P males having significantly more ΔFosB-ir cells compared to sexually naive T@P males. There were no significant differences in ΔFosB-ir cells as a function of sexual experience within NoT@P males. Bars represent mean (±SEM); numbers on bars indicate sample size. +Interaction between pubertal testosterone and sexual experience, *p* ≤ 0.05.

The subregions of the mPfC were traced according to the Morin and Wood (2001) hamster atlas (Fig. 4*A*) as shown by a representative microphotograph in Figure 4*B*. Sexual experience led to a significant increase in ΔFosB expression in the IL of the mPfC only in T@P males (Fig. 4*C*,*D*). In contrast, sexual experience did not increase ΔFosB expression in the Cg1 or PrL in either T@P or NoT@P males (Fig. 4*D*). Although the interaction between pubertal testosterone and experience was not statistically significant within the PrL (*p* = 0.122), experience appeared to increase ΔFosB expression in the PrL in T@P, but not NoT@P, males.

**Figure 5.**
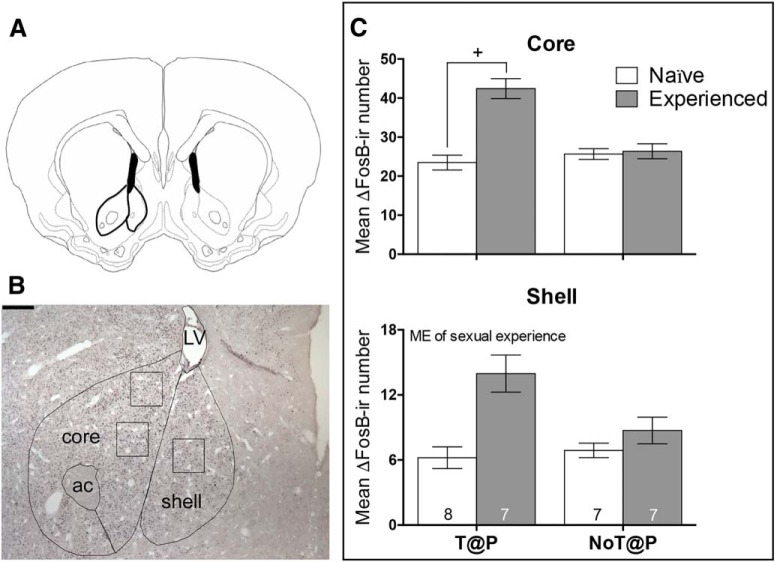
Number of ΔFosB-ir cells in the NAc core and shell is dependent on pubertal testosterone and sexual experience. ***A***, Brain atlas (Morin and Wood, 2001) representation of a coronal section containing the NAc. ***B***, Photomicrographs of drawn contours of the NAc onto immunohistochemically-treated tissue sections. The NAc included the shell and core. LV = lateral ventricle; ac = anterior commissure. Scale bar = 250 µm. ***C***, In the core, there was an interaction between pubertal testosterone and sexual experience on ΔFosB-ir cells with sexual experienced T@P males having significantly more ΔFosB-ir cells compared to sexually naive T@P males. There were no significant differences in ΔFosB-ir cells as a function of sexual experience within NoT@P males. In the shell, there was a main effect (ME) of sexual experience on ΔFosB-ir cells with sexually experienced males having more ΔFosB-ir expression compared to sexually naive males. Bars represent mean (±SEM); numbers on bars indicate sample sizes. +Interaction between pubertal testosterone and sexual experience, *p* ≤ 0.05.

The shell and core of the NAc were also traced according to the Morin and Wood (2001) hamster atlas (Fig. 5*A*) with a representative microphotograph shown in Figure 5*B*. Sexual experience significantly increased ΔFosB expression in the NAc core only in T@P males (Fig. 5*C*). Sexual experience led to a significant increase in ΔFosB expression in NAc shell (Fig. 5*C*); however, pubertal testosterone did not affect ΔFosB expression in the shell nor was there an interaction. Similar to the PrL, although the interaction between pubertal testosterone and experience was not statistically significant within the shell (*p* = 0.115*)*, experience appeared to increase ΔFosB expression in the shell in T@P, but not NoT@P, males.

### Experiment 2: overexpression of ΔFosB in the IL restores social proficiency in NoT@P males (Table 4)

**Figure 6.**
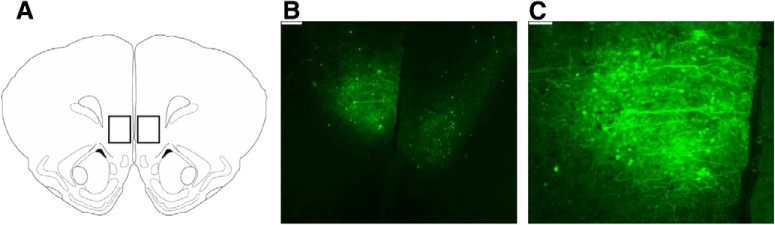
Visualization of GFP to verify injection site and extent of infected cells in the IL. ***A***, Boxes of representative injection sites in the IL for NoT@P-ΔFosB males over coronal atlas diagram (Morin and Wood, 2001). ***B***, Photomicrograph of GFP overexpression in a NoT@P-ΔFosB male; scale bar = 250 µm. ***C***, Photomicrograph of GFP overexpression in a NoT@P-ΔFosB male; scale bar = 100 µm.

**Figure 7.**
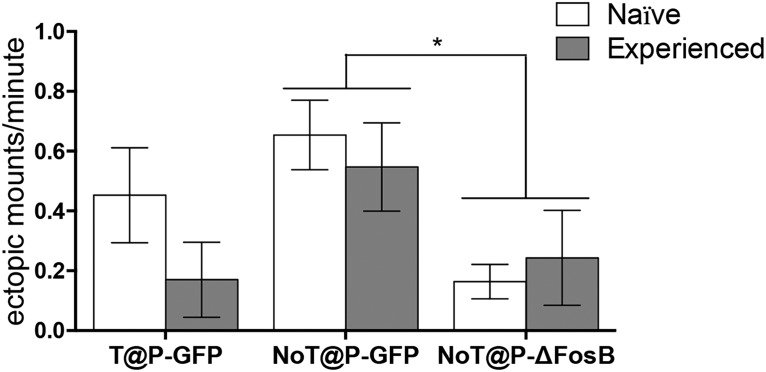
Overexpression of ΔFosB in the IL decreases the rate of ectopic mounting in NoT@P males. NoT@P-ΔFosB males (*n* = 6) had significantly less ectopic mounts per minute compared to NoT@P-GFP males (*n* = 9). T@P-GFP males (*n* = 7) did not differ from either group in rate of ectopic mounting. Bars represent mean (±SEM). *Main effect of experimental group, *p* ≤ 0.05.

Placement of the injection site and extent of transduced cells in the IL was verified using fluorescence microscopy (Fig. 6). Overexpression of ΔFosB in the IL decreased the overall rate of ectopic mounting (Fig. 7), with NoT@P-ΔFosB males displaying less ectopic mounts per minute than NoT@P-GFP males. Sexual experience did not affect the rate of ectopic mounting, nor was there an interaction between experimental group and sexual experience. Interestingly, sexually naive NoT@P-ΔFosB males had similar rates of ectopic mounting as sexually experienced T@P males, suggesting that they become sexually proficient within a single sexual encounter.

**Figure 8.**
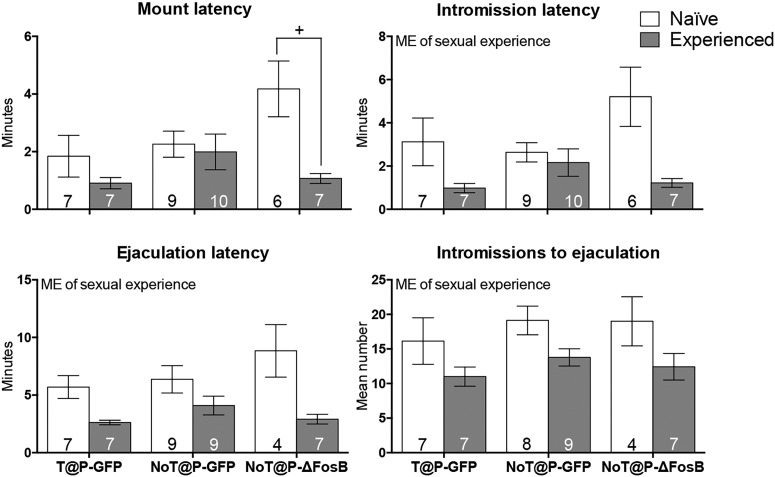
The effects of ΔFosB overexpression in the IL and sexual experience on latency to mount, intromit, and ejaculate and number of intromissions to ejaculation. For mount latency, there was a pubertal testosterone × sexual experience interaction with sexually naive NoT@P-ΔFosB males having a longer latency to mount compared to sexually experienced NoT@P-ΔFosB males. This effect of sexual experience was not found in T@P-GFP or NoT@P-GFP males. For intromission latency and ejaculation latency, there was a main effect of sexual experience with sexually experienced males having shorter latencies to mount and intromit compared to sexually naive males. For intromissions to ejaculate, there was a main effect (ME) of sexual experience with sexually experienced males having less intromissions to achieve ejaculation compared to sexually naive males. Bars represent mean (±SEM); numbers on bars indicate sample size. +Interaction between experimental group and sexual experience, *p* ≤ 0.05.

Sexual experience significantly reduced the latency to intromit, latency to ejaculate, and number of intromissions to reach ejaculation (Fig. 8), independent of overexpression of ΔFosB in the IL, and with no interactions. There was an interaction between sexual experience and overexpression of ΔFosB in the IL for mount latency due to sexual experience decreasing the latency to mount in NoT@P-ΔFosB males, but not in GFP control males (Fig. 8). To assess region specificity, behaviors of the three NoT@P-ΔFosB males with misplaced injections were compared with those of NoT@P-ΔFosB males with accurately placed injections. Due to the low sample size and variable brain region hits, these data were not analyzed statistically, but overall NoT@P-ΔFosB males with misplaced injections did not show evidence of social proficiency similar to that seen in NoT@P-ΔFosB males with accurately placed injections (data not shown).

### Experiment 3: both sexual experience and overexpression of ΔFosB increase spine density in the IL (Table 5)

**Figure 9.**
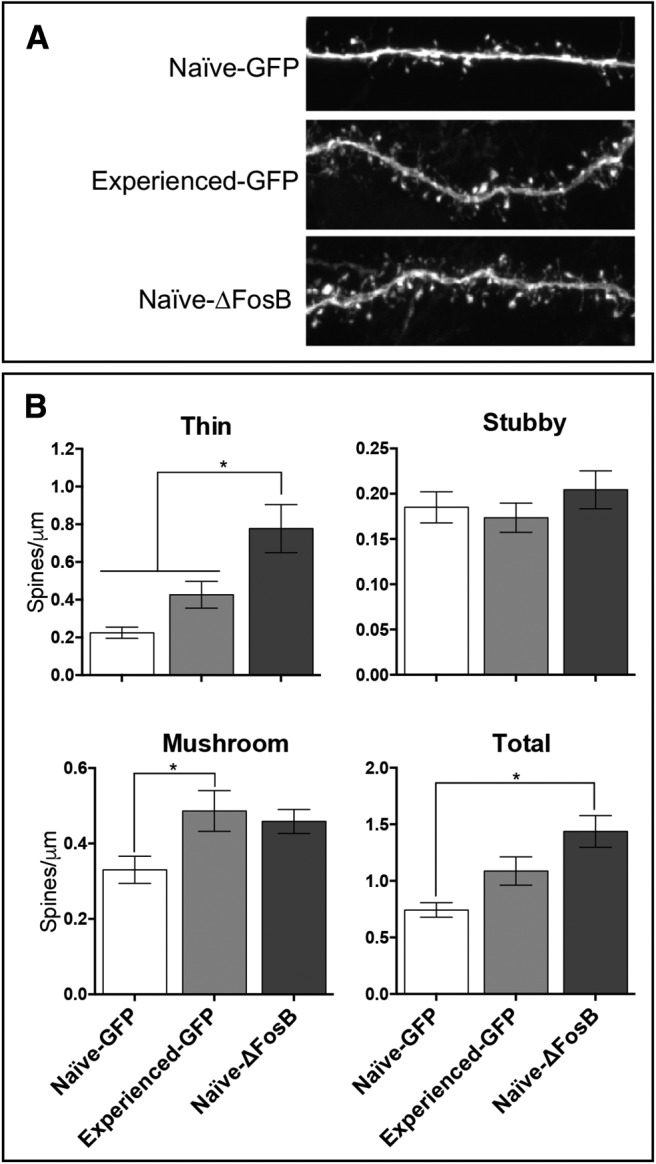
Both sexual experience and ΔFosB increase dendritic spines in vmPFC. ***A***, AAV-GFP was injected into the IL of naive (*n* = 22) or sexually experienced (*n* = 21) males, and AAV-GFP-ΔFosB was injected into the IL of naive males (*n* = 22). Immunofluorescence using a GFP antibody reveals spines of IL pyramidal neurons in all three groups. ***B***, Thin spine density was increased by ΔFosB overexpression. Stubby spine density was not affected by either sexual experience nor ΔFosB overexpression. Mushroom spine density increased with sexual experience and showed a trend to increase by ΔFosB overexpression. Overall, total spine density was increased by ΔFosB overexpression. Bars represent mean (±SEM). *Main effect of experimental group, *p* < 0.05.

Overexpression of ΔFosB in the IL increased thin dendritic spine density (Fig. 9*B*), with naive-ΔFosB males having significantly more thin spines compared to both groups of GFP males. There was no effect of sexual experience or overexpression of ΔFosB in the IL on stubby spines (Fig. 9*B*). Sexual experience significantly increased mushroom spine density (Fig. 9*B*), with experienced-GFP males having more mushroom spines compared to naive-GFP males. Overexpression of ΔFosB in the IL increased total dendritic spine density (Fig. 9*B*), with naive-ΔFosB males having significantly more total spines compared to naive-GFP control males and experienced-GFP males not differing significantly from either group. Thus, we found that males with overexpressed ΔFosB in the IL had a significant increase in total spine density driven by a significant increase in thin spines and a (nonsignificant) increase in mushroom spines.

## Discussion

These studies provide evidence that during puberty, testosterone is necessary for experience-dependent induction of ΔFosB in the IL, thereby programming sexual proficiency in adulthood, possibly through modulation of synaptic lability. We first replicated our previous finding that pubertal testosterone is required for male hamsters to display behavioral adaptations with sexual experience (i.e., social proficiency). We then showed that the presence of testosterone during puberty is necessary for social experience-dependent increases in ΔFosB in both the ventral mPFC and NAc core, providing a correlation between dysregulation of ΔFosB and impairment in social proficiency. Next, we demonstrated that overexpression of ΔFosB in the IL is sufficient to restore a socially proficient phenotype in males that lacked testosterone during puberty. In addition, both ΔFosB and sexual experience increase the density of dendritic spines on IL neurons, albeit in different patterns, suggesting a potential mechanism by which pubertal testosterone programs experience-dependent neural and behavioral adaptations in adulthood. To our knowledge, this is the first report to implicate ΔFosB as a key player in experience-dependent neural and behavioral plasticity that is hormonally programmed during a sensitive period of social development, providing new mechanistic insight into the development of social cognition during puberty and adolescence.

In the present study, we found that induction of ΔFosB in the IL occurred after sexual experience in only T@P males, suggesting a possible site of action in which pubertal testosterone programs the ability to inhibit maladaptive behaviors. Because ΔFosB is induced by persistent neuronal activity (Robison and Nestler, 2011), these data may indicate that the IL is preferentially activated by sexual experience in T@P males, and is not activated to the same extent in NoT@P males. This suggests that pubertal testosterone may rewire the brain to allow increased excitatory input (or less inhibitory input) to the IL, which then regulates behavioral inhibition during social behaviors. Substantial projections from the ventral hippocampal formation (CA1 and subiculum, specifically) and the basolateral amygdala to the IL may contribute to the formation of social memories and social learning through reward-related experience, both of which are required for adaptive behavioral flexibility (Hoover and Vertes, 2007; Wassum and Izquierdo, 2015; Hegde et al., 2016). Indeed, ovarian hormones during puberty organize inhibitory transmission in the Cg1 region of the mPfC to program behavioral flexibility in non-social learning tasks in mice (Piekarski et al., 2017). Thus, the notion that pubertal hormones rewire the brain to fine-tune behavioral inhibition is not unique to testicular hormones and social proficiency, and generalizes to ovarian hormones and other prefrontal-dependent forms of behavioral flexibility across species.

The ventral mPfC, which includes the IL and PrL, is critical for behavioral inhibition in sexual contexts. Male rats with ventral mPfC lesions continue to mate with females even when sexual behavior is paired with aversive consequences (Davis et al., 2010). Furthermore, ΔFosB is integral in inducing neural plasticity in response to both natural and drug rewards by regulating gene expression (Nestler et al., 1999; McClung et al., 2004; Pitchers et al., 2013). Therefore, NoT@P males may not have the capacity for behavioral inhibition as a result of lacking this crucial ΔFosB-driven plasticity within the IL. Indeed, we found that NoT@P males overexpressing ΔFosB show low rates of ectopic mounting overall compared to control NoT@P males. Although NoT@P-ΔFosB males did not show a decrease in ectopic mounting with sexual experience, this is likely due to floor effect as they had very low rates even when sexually naive. In fact, sexually naive NoT@P-ΔFosB males had ectopic mounting rates similar to sexually experienced T@P males, suggesting that ΔFosB overexpression reduces this maladaptive behavior even in the absence of experience.

Our group and others have found that overexpression of ΔFosB causes an increase in immature dendritic spine number in medium spiny neurons of the NAc (Maze et al., 2010; Grueter et al., 2013; Robison et al., 2013) as well as in hippocampal pyramidal neurons (Eagle et al., 2015). Therefore, we hypothesized that ΔFosB might exert its effects on social proficiency through alterations in the number and structure of dendritic spines on IL pyramidal neurons. Indeed, we found that both sexual experience and overexpression of ΔFosB significantly increased the density of spines on IL pyramidal neurons, with sexual experience increasing mature mushroom spines and overexpression of ΔFosB increasing thin and total spines. These results are consistent with previous work: sexual experience in female hamsters increases NAc spine density (Staffend et al., 2014); in other rodents, sexual experience in males increases mPfC pyramidal neuron spine density (Glasper et al., 2015), early-life stress reduces both social interaction and mPfC spines (Farrell et al., 2016), and antidepressant treatment increases both dendritic spine number and ΔFosB expression in PfC (Li et al., 2010; Vialou et al., 2014). One intriguing potential explanation for these findings is that sexual experience increases ΔFosB expression driving an initial increase in thin spines, while consolidation of memories encoding learned sexual behavior is accompanied by a return to basal ΔFosB levels and a maturation of spines into a mushroom shape. In experiments in which ΔFosB expression is maintained virally without sexual experience, as we have done here, new immature spines are constantly generated, but no learning occurs to cause them to form into mature mushroom spines. The gene targets of ΔFosB in the mPfC are largely unknown, though in other brain regions, including NAc, it regulates the expression of multiple genes critical for spine dynamics and glutamatergic synapse function, including CaMKIIα (Robison et al., 2013) and GluA2 (Kelz et al., 1999; Vialou et al., 2010). These studies, together with the current findings, suggest that ΔFosB increases the potential for synaptic plasticity via the formation of immature spines, and that behavioral experience is needed to promote conversion of labile thin spines into more stable mushroom spines.

Sexual proficiency is gained through learning from experience and making behavioral adaptations in part through the inhibition of behaviors that become maladaptive with experience, such as ectopic mounting. The major behavioral difference found between T@P and NoT@P males was rate of ectopic mounting. NoT@P males showed initial high rates of ectopic mounting and, even after sexual experience, continued to show high rates. Conversely, sexually naive T@P males had lower rates of ectopic mounting compared to NoT@P males, and also showed a decrease in ectopic mounting with sexual experience. Being able to inhibit behaviors that are maladaptive is imperative to successful social interactions. Therefore, these data indicate that pubertal testosterone programs the ability to inhibit behaviors that are not rewarding or advantageous, and thus, maladaptive.

Here, we provide evidence that pubertal testosterone organizes the ability to inhibit inappropriate behavior. However, there are some limitations to the present set of experiments. Although the male Syrian hamster is an excellent model to study both sexual reward and sexual proficiency, hamsters are a solitary species. Thus, the generalizability of our results to more social species is limited. Perhaps pubertal testosterone plays a bigger role in organizing the brain in solitary species compared to social species, as solitary animals do not have as much exposure to social experience to help shape the brain. Experiments that address this issue in a more social species, such as rats, would be useful. Additionally, sexual proficiency is only one example of social cognition, and it remains to be seen whether the principles we have derived from our studies on sexual proficiency generalize to other types of social proficiency and cognition. We have shown similar deficits in social cognition during male-male agonistic encounters of NoT@P males (Schulz et al., 2006; De Lorme and Sisk, 2013). Thus, we hypothesize that the social ineptness of NoT@P males is not unique to a specific social context, but reflects a more global dysfunction in the expression of context-appropriate social behavior. Finally, it is worth noting that we also found an increase in ΔFosB in the NAc core after sexual experience in T@P, but not NoT@P, males, and it is possible that a similar increase in ΔFosB and/or dendritic spine density occurred in other regions that we did not investigate. Therefore, although we have shown that overexpression of ΔFosB in ventral mPFC is sufficient to induce a socially proficient behavioral phenotype, additional experiments are needed to show that an increase in ΔFosB in the IL is necessary for social proficiency to be acquired.

Given that social contexts are ever changing, social proficiency is a fundamental asset in adulthood because it increases the probability of successful social interactions. We show here that, in males, exposure to testosterone during puberty is critical for the ability to make behavioral adjustments with sexual experience. Without pubertal testosterone, males show neither the neural (increase in ΔFosB expression) nor behavioral (decrease in ectopic mounts) adaptations to sexual experience in adulthood that are observed in typically developing males. We also show that, in males who were exposed to testosterone during puberty, the overexpression of ΔFosB in IL increases the density of immature spines and sexual experience increases the density of mature spines, suggesting a mechanism by which pubertal testosterone programs neural and behavioral plasticity in adulthood. We speculate that in the absence of testosterone during puberty, adult males fail to acquire social proficiency as a result of the lack of ΔFosB-induced formation of immature spines in the IL that could mature into functional glutamatergic synapses with social experience. That is, without the potential for neural plasticity in executive control brain regions, there is less potential for experience-dependent behavioral plasticity. The sites and mechanisms of action for hormonal programming of brain and behavior during puberty are largely unknown, and thus, our findings prompt new avenues of investigation to advance understanding of adolescent maturation of social cognition.

## Synthesis

Reviewing Editor: Margaret McCarthy, Univ of Maryland School of Medicine Decisions are customarily a result of the Reviewing Editor and the peer reviewers coming together and discussing their recommendations until a consensus is reached. When revisions are invited, a fact-based synthesis statement explaining their decision and outlining what is needed to prepare a revision will be listed below. Note: If this manuscript was transferred from JNeurosci and a decision was made to accept the manuscript without peer review, a brief statement to this effect will instead be what is listed below.

The authors have done an excellent job of responding to the previous reviews.
